# Genome-Wide Association Study for Autism Spectrum Disorder in Taiwanese Han Population

**DOI:** 10.1371/journal.pone.0138695

**Published:** 2015-09-23

**Authors:** Po-Hsiu Kuo, Li-Chung Chuang, Mei-Hsin Su, Chia-Hsiang Chen, Chien-Hsiun Chen, Jer-Yuarn Wu, Chung-Jen Yen, Yu-Yu Wu, Shih-Kai Liu, Miao-Chun Chou, Wen-Jiun Chou, Yen-Nan Chiu, Wen-Che Tsai, Susan Shur-Fen Gau

**Affiliations:** 1 Institute of Epidemiology and Preventive Medicine, College of Public Health, National Taiwan University, Taipei, Taiwan; 2 Research Center for Genes, Environment and Human Health, National Taiwan University, Taipei, Taiwan; 3 Department of Nursing, Cardinal Tien Junior College of Healthcare & Management, I-Lan, Taiwan; 4 Department of Psychiatry, Chang Gung Memorial Hospital-Linkou, Taoyuan, Taiwan; 5 Department and Graduate Institute of Biomedical Sciences, Chang Gung University, Taoyuan, Taiwan; 6 Institute of Biomedical Sciences, Academia Sinica, Taipei, Taiwan; 7 Department of Internal Medicine, National Taiwan University Hospital and College of Medicine, Taipei, Taiwan; 8 Department of Child and Adolescent Psychiatry, Taoyaun Psychiatric Center, Ministry of Health and Welfare, Taoyuan, Taiwan; 9 Department of Child Psychiatry, Kaohsiung Chang Gung Memorial Hospital and Chang Gung University College of Medicine, Taoyuan, Taiwan; 10 Department of Psychiatry, National Taiwan University Hospital and College of Medicine, Taipei, Taiwan; University of Jaén, SPAIN

## Abstract

**Background:**

Autism spectrum disorder (ASD) is a neurodevelopmental disorder with strong genetic components. Several recent genome-wide association (GWA) studies in Caucasian samples have reported a number of gene regions and loci correlated with the risk of ASD—albeit with very little consensus across studies.

**Methods:**

A two-stage GWA study was employed to identify common genetic variants for ASD in the Taiwanese Han population. The discovery stage included 315 patients with ASD and 1,115 healthy controls, using the Affymetrix SNP array 6.0 platform for genotyping. Several gene regions were then selected for fine-mapping and top markers were examined in extended samples. Single marker, haplotype, gene-based, and pathway analyses were conducted for associations.

**Results:**

Seven SNPs had p-values ranging from 3.4~9.9*10^−6^, but none reached the genome-wide significant level. Five of them were mapped to three known genes (*OR2M4*, *STYK1*, and *MNT*) with significant empirical gene-based p-values in *OR2M4* (p = 3.4*10^−5^) and *MNT* (p = 0.0008). Results of the fine-mapping study showed single-marker associations in the *GLIS1* (rs12082358 and rs12080993) and *NAALADL2* (rs3914502 and rs2222447) genes, and gene-based associations for the *OR2M3-OR2T5* (olfactory receptor genes, p = 0.02), and *GLIPR1/KRR1* gene regions (p = 0.015). Pathway analyses revealed important pathways for ASD, such as olfactory and G protein–coupled receptors signaling pathways.

**Conclusions:**

We reported Taiwanese Han specific susceptibility genes and variants for ASD. However, further replication in other Asian populations is warranted to validate our findings. Investigation in the biological functions of our reported genetic variants might also allow for better understanding on the underlying pathogenesis of autism.

## Introduction

Autism spectrum disorder (ASD) is a severe neurodevelopmental disorder characterized by various degree of abnormal language/communication and social reciprocity, and restricted repetitive behaviors/interests [1,2] and resulting in great impacts on individuals, families, and society. Since the contribution of genetic factors to the susceptibility of ASD has been extensively documented with high heritability (80~90%) [3], this disorder has been prioritized for molecular genetic studies [4]. Previous efforts in gene mapping for ASD often produced inconsistent results, and many of the prior reported risk genetic variants are rare—only accounting for a small fraction of ASD cases. Some—particularly structural variants—are *de novo* mutations that do not directly contribute to the high heritability of ASD [5] such as exonic deletions at *NRXN1* and gene duplications encompassing *UBE3A* in the 15q11–q13 region [6]. It is believed that highly rare penetrant mutations and common variants with multiplicative effects all contribute to causing a complex disorder like autism. Thus, many rare variants and chromosomal deletions have been identified for ASD although susceptible common variants for ASD are still largely unknown [7].

Recently, several genome-wide association (GWA) studies of ASD have suggested a number of common genetic variants associated with the risk of ASD [8–12]. Weiss et al. (2009) reported signals on chromosome 5p15, and their results from association testing implicated that *SEMA5A* is an autism-susceptible gene. In addition, several single nucleotide polymorphisms (SNPs) with p-values ranging from 10^−4^ to 10^−6^ in a novel region on chromosome 5p14.1 were reported to be associated with ASD in 487 Caucasian autism families, and the significance level was boosted to 2*10^−10^ in a meta-analysis using larger autism family and case-control cohorts in this chromosomal region [9,11]. Kerin et al. (2012) [13] further identified an antisense non-coding RNA, *MSNP1AS* in this region, which is overexpressed in postmortem cerebral cortex of individuals with ASD. A study of ASD in the US samples also had significant findings in the regions of 5q21.1 and 15q22.1–q22.2. Markers in the 5q21.1 region does not map to known genes, but markers in the 15q22 region are located within the *SLTM* gene, which encodes for estrogen-induced transcription modulator [10]. With more than four thousand individuals, the Autism Genome Project (AGP) consortium identified associations in the *MACROD2* gene (rs4141463) for which the biological function was largely unknown [8]. A large follow-up association study in Europe with more than one thousand cases and thirty-five thousand controls failed to replicate the association of the marker rs4141463 in the *MACROD2* gene [14]. Later, using a combined sample with 2705 families in AGP, another three markers in this gene region again showed associations at the level of 10^−7^ [15].

It is apparent that consistent and replicated results have not been seen across these aforementioned large-scale GWA studies with thousands of subjects, which could be partially attributed to genetic heterogeneity. In addition, pooling samples from different countries (even though they are all Caucasian populations) might introduce noises due to ethnic heterogeneity. Thus far, there have been very few systematic examinations of the associations for ASD using the GWA strategy in the Asian populations [16]. We also noticed that none of the previously investigated common loci conveyed a large disease risk, consistent with the hypothesis that many genes—each with a small effect—are involved in the etiology of ASD [15,17]. Thus, to identify common genetic variants for the risk of ASD in Taiwanese Han population, the current study applied a two-stage GWA approach to facilitate gene findings in complex traits [18]. During the first stage, we employed a GWA screening for ASD patients and healthy controls. A follow-up fine-mapping study was then conducted in an extended sample to identify promising genes and markers for ASD.

## Materials and Methods

### Participants

We conducted a family study of ASD to recruit probands with ASD, their biological parents, and siblings. Index ASD probands were enrolled from the outpatient clinics of one university hospital, one private medical center, one psychiatric hospital in Northern Taiwan, one private medical center in Southern Taiwan, primary and secondary schools, and early intervention centers nationwide. Patients were clinically diagnosed with autistic disorder or Asperger’s disorder according to the DSM-IV diagnostic criteria [19] and confirmed by qualified child psychiatrists using the Chinese version of the Autism Diagnostic Interview-Revised (ADI-R). The ADI-R is a standard, comprehensive, and semi-structured interview that focuses on three symptom domains of the DSM-IV and ICD-10 [20] diagnostic criteria, including deficits in reciprocal social interaction, impaired verbal, and non-verbal communication as well as restricted, repetitive, and stereotyped patterns of behaviors [21]. The Chinese version of the ADI-R has been approved by the Western Psychological Services in 2007, and is widely used in ASD research (e.g., [22–26]) in Taiwan.

Probands underwent complete assessments of environmental, perinatal, developmental, medical, and clinical conditions in addition to neuropsychological tests. Parents also reported on the Chinese version of the social communication quotient regarding the three autistic core features of participants [27]. All the subjects were Taiwanese Han. For more details on the sample recruitment and assessment, please refer to Lin et al., (2013) [25].

The study design and subjects information of this study are illustrated in S1 Fig. For the first stage of GWA screening, we enrolled 1,164 subjects from 393 families (probands aged 9.1±3.99 years, male 88.6%). Probands diagnosed with fragile X and Rett’s disorder based on DNA testing or clinical features were excluded from the recruitment [28]. Additionally, probands with previously identified chromosomal structural abnormality associated with autism or had any other major neurological or medical conditions were also excluded [28]. The Research Ethics Committee of four research sites approved this study [ClinicalTrials.gov number, NCT00494754]. Written informed consent was obtained from all the parents and the majority of the participants (child asset if written informed consent was not applicable) after the procedures of the study were fully explained and confidentiality was ensured.

Healthy controls were from the Han Chinese Cell and Genome Bank (HCCGB) in Taiwan. In brief, a three-stage probability clustering sampling through the registry of all 329 non-aboriginal townships or city districts was implemented to obtain representative samples. Complete bio-specimen and questionnaire data (with a focus on ethnicity and medical history) were also collected for 3,380 adult individuals. Written informed consent was obtained from all participants. The recruitment details were documented elsewhere [29]. Of the 3,380 controls, 1,139 controls with a Taiwanese Han ancestry, who were found to have no definite diagnosis of any major medical or mental illnesses, underwent genotyping at the genome-wide level and were treated as controls in the GWA screening stage.

To extend our original samples in GWA screening, we continuously recruited additional 282 ASD cases from clinical settings aforementioned. Additional 480 unrelated controls were also recruited, including 120 male youth controls aged 8–18, 120 young men aged 18–22, and 240 healthy controls from the HCCGB in Taiwan [29], resulting in total 597 ASD cases and 1595 controls. The 120 male youth controls and their parents were clinically assessed by the corresponding author to confirm that they did not have any of the following psychiatric disorders: ASD, attention-deficit hyperactivity disorder, anxiety disorders, mood disorders, or major psychosis. The 120 young men were screened by the Chinese versions of the Adult Self Report Inventory-4 [30] to exclude DSM-IV psychopathology and the Autism Spectrum Quotient [31] to exclude the Autistic trait.

### Genotyping and quality controls

Genomic DNA was extracted from peripheral white blood cells using the QIAamp DNA Blood Mini kit (QIAGEN Inc., Valencia, CA, USA). The measurement of quantity was performed by the PicoGreen method and traditional optical spectrophotometry to normalize to a concentration of approximately 50 ng/uL or greater. We used the Affymetrix Genome-Wide Human SNP6.0 array to obtain genotyping data of around one million each SNP (single nucleotide polymorphism) marker and CNV (copy number variation) probe (Affymetrix Inc., Santa Clara, CA, USA). Genotype calling was determined by the Birdseed algorithm using default parameters. Quality control of genotype data was performed by examining several summary statistics. Autosomal SNPs were excluded for further analysis if they were monomorphic, had a minor allele frequency (MAF) of less than 5%, had total call rate less than 98%, or significantly departed from the Hardy-Weinberg equilibrium in the control samples (P-value<10^−4^). A total of 546,171 SNPs were retained for the association analysis.

341 probands with autism and 1139 controls were successfully genotyped with the SNP6.0 array. Probands and controls were checked for kinship relationships, and participants who had a call rate less than 99% were removed to reduce the chance of genotyping errors due to poor DNA quality. Detection of possible population stratification that might influence association analysis was carried out using multiple-dimensional scaling in PLINK (http://pngu.mgh.harvard.edu/~purcell/plink/), and no individual was removed after combining our samples with HapMap samples to detect outliers. After removing individuals who had kinship relationships to the level of kinship coefficient greater than 1/8 (6 in cases and 24 in controls) and had previously identified chromosomal structural abnormality associated with autism (20 cases), 315 patients with autism and 1115 controls were retained in the screening stage of the GWA analysis, including 819 males and 611 females. The total genotyping rate in these remaining samples was 99.76%.

### Fine mapping for selected gene regions from the GWA study

Markers on the top of the significant findings from the first-stage of GWA screening were subjected to selection into the fine-mapping stage with extending samples. First, markers in the GWA study that had association p-value less than 5*10^−5^ were mapped to known genes database. For SNPs that fell into a known gene, a fine-mapping strategy was employed to achieve a better coverage rate for the mapped genes in a subset of overall samples with 480 independent cases and 480 controls. A genomic region, including 5 kb up-and down-stream of the gene, was obtained from the NCBI Human Genome Build 36. SNPs data with MAF > 0.01 within each gene region from the HapMap Chinese in Metropolitan Denver was retrieved, and we used SNPHAP (https://www-gene.cimr.cam.ac.uk/staff/clayton/software/) to reconstruct haplotypes for each gene. We intended to genotype minimum index SNPs that represent the maximum haplotype information and Tagger was used to select tagging SNPs for identified loci (de Bakker et al. 2005). In total, we selected seven gene regions for fine-mapping study, including *OR2M3-OR2T5*, *STYK1*, *GSTZ1*, *GLIPR1-KRR1*, *DDX19A-DDX19B*, *SGSM2-MNT*, *KCNE1*, and retrieved 76 SNPs for these loci.

We used the Illumina custom genotyping array with multiplex assays in this stage. Genotyping was performed by Illumina GoldenGate according to the manufacturer’s protocol with 500ng of sample DNA per assay. All pre-PCR processing was performed using a TECAN liquid handling robot running Illumina protocols. Arrays were imaged using an Illumina Beadstation GX500, and we used the GenCall v6.2.0.4 and GTS Reports software v5.1.2.0 (Illumina Inc.) to analyze data. The performance of the initially selected SNP set was validated by the manufacturer and replacements were made where necessary.

Additionally, there were 19 markers located in other genes or the intergenic regions with p-values less than 5*10^−5^ in the GWA analysis, and one marker of a previously reported autism associated gene (*MACROD2*, [8]) with a p-value of 9.95*10^−5^. These 20 markers were also tested in the full sample of 597 ASD cases and 1595 controls.

### Statistical analyses

Initial GWA analysis was carried out by comparing allele/genotype frequencies between 315 cases and 1115 controls at the single marker level, including methods of allele-type, Cochran-Armitage trend test, along with tests of dominant and recessive inheritance models using PLINK [32]. Empirical p-values were obtained with 10^6^ simulations. SNPs with p-values less than 5*10^−5^ were considered to have suggestive associations. Because the results were similar across different genetic models, we reported association results using the allelic model. In addition, multi-point/haplotype analysis was performed using the Haplotype Score Test implemented in haplo.stat, a suite of S-PLUS/R routines for the analysis of indirectly measured haplotypes with 3-point sliding windows across the genome [33]. Haplotype specific associations were evaluated with haplotypes frequency greater than 0.05. Markers that showed suggestive associations were mapped to the known gene database, and gene-based analysis was conducted using the versatile gene-based association study method [34]. To examine the common processes and underlying biological mechanisms among the significant genes (p<0.01) from gene-based analysis, we employed pathway analyses using GO terms for functional annotation and enrichment analysis. The hypergeometric test was performed using the web-based Gene Set Enrichment Analysis (http://www.broadinstitute.org/gsea/index.jsp). A p-value<0.05 after the Bonferroni correction was considered significant.

At the fine-mapping stage, single marker analysis for these markers was similarly performed as aforementioned methods. A gene-based analysis was conducted to analyze the associations between the seven selected gene regions with ASD using PLINK with the default setting (pairwise r^2^ less than 0.5). The empirical p-values of the gene-based test were obtained through 10,000 permutations.

## Results

**Fig 1:**
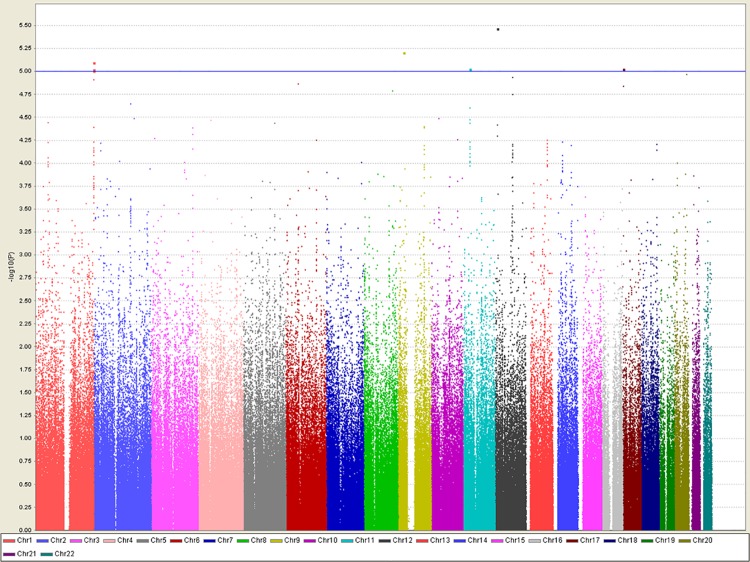
Manhattan plot of genome-wide association analysis. Results of genome-wide association analysis (–log_10_P) are shown in chromosomal order for 546,171 SNPs that were tested for association in the initial sample of 315 ASD cases and 1115 controls. The horizontal line indicates a P-value of 10^−5^.

**Fig 2:**
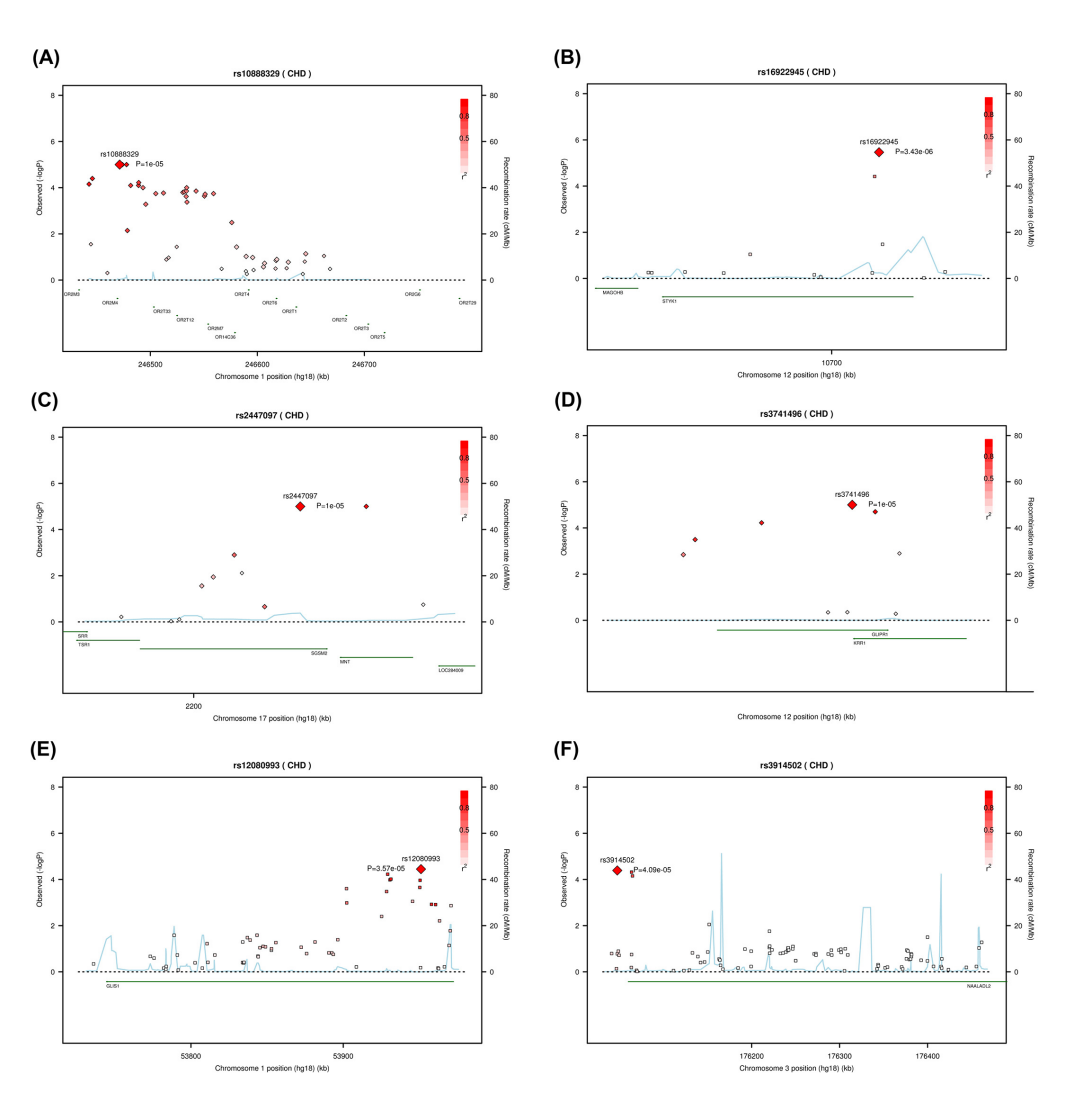
Locus-plot of reported associated gene regions for ASD. (A) *OR2M3_OR2T5*. (B) *STYK1*. (C) *SGSM2_MNT*. (D) *GLIPR1_KRR*. (E) *GLIS1*. (F) *NAALADL2*.

The Manhattan plot of 546,171 SNPs that were tested for the association in the initial GWA sample of 315 ASD cases and 1115 controls is shown in Fig 1. The quantile-quantile plot of the GWA analysis is shown in S2 Fig. The genomic inflation factor, lambda, was 1.023. The primary single point GWA analysis revealed that seven SNPs were associated with ASD with p-values in the range of 3.4~9.9*10^−6^, but none reached the genome-wide significance level. Five out of the seven markers were mapped to three known genes, *OR2M4*, *STYK1*, and *MNT*. We listed markers with p-values less than 5*10^−5^ in Table 1. Table 1 presents the results of the single marker analysis along with the gene-based results for SNPs that were mapped to known genes. The most significant marker, rs16922945 (p = 3.4*10^−6^), is located in the *STYK1* gene (intronic region) on chromosome 12 with an odds ratio (OR) of 1.86 (95% CI, 1.43~2.43). The *STYK1* gene, serine-threonine-tyrosine kinase 1, plays important roles in diverse cellular and developmental processes such as cell proliferation, differentiation, and survival [35]. However, gene-based analysis for the *STYK1* gene was not significant (p = 0.09) in the GWA samples. Three markers were mapped to the *OR2M4* gene with the gene-based p-value of 3.4*10^−5^. Another marker, rs2447097, showed a weak association with ASD (OR = 1.53, p = 9.5*10^−6^). This marker was mapped to both of the *SGSM2/MNT* genes (Small G Protein Signaling Modulator 2; MAX dimerization protein), and the gene-based analysis of the *MNT* gene exhibited association with ASD (p = 0.0008). The rest of the two markers that had p-values less than 1*10^−5^ did not map to any known genes, and OR ranged from 1.5–1.8, including markers rs10966205 on chromosome 9 and rs7933990 on chromosome 11. Locus plots of the three gene regions, *OR2M4*, *STYK1*, and *SGSM2/MNT*, are shown in Fig 2A to 2C.

**Table 1:** Results of single marker and gene-based association analyses in the GWA study.

SNP	CHR	Position	Allele	ASD	Control	Index SNP	LD range (kb)	OR	(95% confidence interval)	P-value	Nearby Genes ^a^	P-value of Gene based analysis
rs12080993	1	54,178,501	A	0.378	0.291	-		1.48	(1.23–1.78)	3.57X10^-05^	*GLIS1*	9.38X10^-04^
rs11204613	1	248,379,155	G	0.119	0.189	rs10888329	31	0.58	(0.45–0.75)	4.06X10^-05^	*OR2M3*	3.90X10^-05^
rs10888329	1	248,404,654	T	0.119	0.196	rs10888329	31	0.55	(0.43–0.72)	8.05X10^-06^	*OR2M4*	3.40X10^-05^
rs6672981	1	248,407,200	C	0.116	0.192	rs10888329	31	0.55	(0.42–0.72)	9.64X10^-06^	*OR2M4*	3.40X10^-05^
rs4642918	1	248,411,057	C	0.116	0.191	rs10888329	31	0.56	(0.43–0.73)	1.24X10^-05^	*OR2M4*	3.40X10^-05^
rs4397683	1	248,411,089	C	0.116	0.192	rs10888329	31	0.55	(0.42–0.72)	9.86X10^-06^	*OR2M4*	3.40X10^-05^
rs3916984	2	157,445,840	T	0.176	0.258	-		0.62	(0.49–0.77)	2.25X10^-05^	*GPD2*	5.79X10^-04^
rs13014164	2	170,271,231	C	0.087	0.045	-		2.04	(1.45–2.87)	3.23X10^-05^	*LRP2/BBS5*	4.93X10^-01^
rs3914502	3	174,564,602	T	0.124	0.195	rs3914502	16	0.58	(0.45–0.76)	4.09X10^-05^	*NAALADL2*	3.43X10^-02^
rs2222447	3	174,580,826	A	0.140	0.213	rs3914502	16	0.60	(0.47–0.77)	4.81X10^-05^	*NAALADL2*	3.43X10^-02^
rs7697680	4	55,245,541	G	0.115	0.065	-		1.87	(1.38–2.52)	3.41X10^-05^	*PDGFRA*	5.15X10^-01^
rs11741756	5	132,579,659	T	0.175	0.113	-		1.67	(1.31–2.13)	3.64X10^-05^	*FSTL4*	9.45X10^-03^
rs13211684	6	51,175,486	G	0.283	0.201	-		1.56	(1.28–1.91)	1.36X10^-05^		
rs12543592	8	123,511,652	G	0.398	0.496	-		0.67	(0.56–0.81)	1.63X10^-05^		
rs10966205	9	24,095,607	A	0.419	0.322	-		1.52	(1.27–1.83)	6.25X10^-06^		
rs7026342	9	111,280,201	G	0.111	0.063	rs7026342	~1	1.86	(1.38–2.52)	4.13X10^-05^	* *	
rs7030851	9	111,281,164	A	0.111	0.063	rs7026342	~1	1.87	(1.38–2.52)	4.01X10^-05^	* *	
rs10763893	10	33,011,229	A	0.084	0.043	-		2.06	(1.46–2.93)	3.26X10^-05^	*C10orf68*	3.29X10^-02^
rs12366025	11	29,148,890	T	0.184	0.119	rs7933990	65	1.67	(1.31–2.11)	2.49X10^-05^	* *	
rs11030597	11	29,190,528	C	0.184	0.120	rs7933990	65	1.65	(1.30–2.10)	3.35X10^-05^		
rs7933990	11	29,208,679	A	0.181	0.114	rs7933990	65	1.72	(1.35–2.19)	9.40X10^-06^		
rs11030606	11	29,214,412	T	0.181	0.118	rs7933990	65	1.65	(1.30–2.10)	3.65X10^-05^	* *	
rs7953930	12	10,818,182	G	0.183	0.119	rs16922945	~1	1.65	(1.30–2.09)	3.83X10^-05^	*STYK1*	9.18X10^-02^
rs16922945	12	10,819,146	C	0.148	0.085	rs16922945	~1	1.86	(1.43–2.43)	3.43X10^-06^	*STYK1*	9.18X10^-02^
rs3741496	12	75,891,253	C	0.535	0.436	rs3741496	2	1.49	(1.24–1.78)	1.15X10^-05^	*KRR1*	3.50X10^-05^
rs1051446	12	75,894,114	C	0.533	0.437	rs3741496	2	1.47	(1.23–1.76)	1.77X10^-05^	*KRR1*	3.50X10^-05^
rs2447097	17	2,278,064	A	0.351	0.261	rs2447097	15	1.53	(1.27–1.85)	9.45X10^-06^	*MNT*	8.03X10^-04^
rs2447095	17	2,293,420	A	0.344	0.257	rs2447097	15	1.52	(1.26–1.84)	1.45X10^-05^	*MNT*	8.03X10^-04^
rs12479663	20	52,708,752	G	0.146	0.087	-		1.81	(1.38–2.36)	1.08X10^-05^	BCAS1/CYP24A1	3.05X10^-02^

^*a*^ The nearby gene is within 100 kb away

Haplotype analysis revealed several potential regions with p-values ranging from 1.8*10^−4^ to 3.6*10^−6^ (S1 Table); haplotypes in genes *NAALADL2*, *SGSM2/MNT*, and *CYP24A1/BCAS1* showed associations with ASD. For gene-set analysis, we included 314 genes with p<0.01 from the GWA analysis and results are listed in Table 2. We found 14 significant pathways for ASD such as the olfactory signaling pathway, G-protein coupled receptor (GPCR) downstream signaling, cytoplasm, and cell proliferation.

**Table 2:** Pathway annotation for significant genes from gene-based association analyses in the GWA study of autism.

Pathway Name	No. of Gene in Gene Set	No. of Gene in Overlap	p-value	FDR q-value
Reactome_Olfactory signaling pathway	328	18	1.02 X10^-13^	2.83 X10^-10^
KEGG_Olfactory transduction	389	18	1.81 X10^-12^	2.51 X10^-09^
Reactome_GPCR downstream signaling	805	23	2.74 X10^-11^	2.54 X10^-08^
Reactome_Signaling by GPCR	920	24	6.39 X10^-11^	4.43 X10^-08^
GO_Cytoplasm	2131	30	3.93 X10^-07^	2.18 X10^-04^
GO_Nucleus	1430	21	1.31 X10^-05^	5.19 X10^-03^
GO_Cell proliferation	513	12	1.31 X10^-05^	5.19 X10^-03^
GO_Multicellular organismal development	1049	16	9.33 X10^-05^	3.24 X10^-02^
GO_Negative regulation of cellular process	646	12	1.19 X10^-04^	3.68 X10^-02^
GO_Regulation of cell proliferation	308	8	1.82 X10^-04^	4.64 X10^-02^
GO_Negative regulation of biological process	677	12	1.84 X10^-04^	4.64 X10^-02^
GO_Macromolecule localization	235	7	2.02 X10^-04^	4.66 X10^-02^
Reactome_Signaling by insulin receptor	108	5	2.25 X10^-04^	4.80 X10^-02^
GO_Cytoplasmic part	1383	18	2.46 X10^-04^	4.87 X10^-02^

Note: FDR (False Discovery Rate)

In the fine-mapping stage, seven subjects had a genotyping call rate less than 0.8 and were excluded from analysis. For the seven selected gene regions, one region (*GSTZ1* gene) did not have multiple SNPs after the quality control of markers was executed, and was excluded from set-based analysis. The association results for the rest of the six regions are displayed in Table 3. Two regions had empirical *p*-values less than 0.05, the *OR2M3-OR2T5* (olfactory receptor, family 2 genes) region (*p* = 0.02) and *GLIPR1/KRR1* (Glioma pathogenesis related protein 1; KRR1 small subunit processome component homolog) gene region (*p* = 0.015). Locus plot of the *GLIPR1/KRR1* gene regions is shown in Fig 2D.

**Table 3:** Set-based analysis in six gene regions in the fine-mapping study.

Chromosome	Gene region	No. of SNP	P__EMP_ ^a^
1	*OR2M3-OR2T5*	7	0.022
12	*STYK1*	15	0.355
12	*GLIPR1/KRR1*	3	0.015
16	*DDX19A-DDX19B*	5	0.418
17	*SGSM2/MNT*	19	0.596
21	*KCNE1*	21	0.959

^a^P__EMP_: empirical p-values derived from 10,000 permutations, with dominance model for *OR2M3-OR2T5*, and additive model for *GLIPR1/KRR1* gene region

Twenty markers were genotyped in the overall samples and results are listed in S2 Table. The results revealed that 13 out of the 20 markers had association p-values less than 1*10^−3^. Four of them were mapped to known genes, including *GLIS1* (rs12082358 and rs12080993) and *NAALADL2* (rs3914502 and rs2222447). Locus plots of the two gene regions, *GLIS1* and *NAALADL2*, are shown in Fig 2E and 2F. Another two markers had genes nearby (around 20 kb away), including *GPD2* (rs3916984) and *BCAS1/CYP24A1* (rs12479663).

## Discussion

Despite high heritability of ASD, very few genes are consistently recognized as the common susceptible loci for ASD. While rare and functional mutations are considered to exert larger effects than common genetic variants do [6, 34], the population attributable effect might be small. On the other hand, the average individual effect size of common genetic variants for the risk of ASD is usually small. To what extent these variants influence the liability of ASD may depend on our ability to identify all possible common susceptible loci. Interestingly, a recent study evaluated the genetic contribution of common variation throughout the genome. Their results suggest that common genetic polymorphism exerts substantial additive genetic effects that explain 40% of heritability in ASD [17]. These findings again emphasize the complex nature of ASD for its underlying genetic architecture and the necessity to capture more genetic variants to building up a biological network for ASD.

Numerous chromosomal regions and genetic loci are reported to be associated with ASD with very few robust findings (for more details, please refer to the review article, [36]). In addition, results from different analytic approaches (e.g. commonly used SNP-based or gene-based analyses) may represent different biological meanings. In addition, markers in the intergenic regions are often not included in such analysis and result in information loss [Li et al., 2012]). So far, only a handful of GWA studies have been conducted for ASD or autism related quantitative traits (e.g. language delay) [8–12,16]. Few loci with moderate effects were identified. For instance, the associations of *MACROD2* showed borederline associations (2*10^−7^) with ASD when samples are enlarged in the AGP [8, 15], and similar findings are shown in the current study, with a p-value of 9.95*10^−5^ in the screening samples and the significance level reduced in the combined samples. On the other hand, non-replication is not uncommon in genetic association studies of complex psychiatric traits. With phenotypic heterogeneity, diverse linkage signals, and risk loci identified in various studies, etiological and genetic heterogeneity has been suggested across subsyndromes and different populations. Studies reported that higher rates of loss of function mutations are associated with greater language and behavior impairments, and lower IQ [Robinson et al. 2014]; diverse sub-phenotypic manifestations of CNVs are reported in ASD samples of European ancestry [37].Two previous GWA studies are conducted for autism-related traits in Asian populations. Cho et al., [16] genotyped 42 ASD trios in Koreans for language delay. While no genome-wide significant findings were reported, two markers in chromosome 11 were suggested to be associated with language delay in ASD. One study in northeastern Chinese samples reported weak associations with two other gene regions [38]. To our best knowledge, the present study reported the first GWA scan for common genetic variants in the diagnosis of ASD with a moderate sample size in Asia. Our results suggest the involvement of several novel genes in the risk of developing ASD, particularly the olfactory receptor gene family, *GLIPR1/KRR1*, *GLIS1*, and *NAALADL2*.

In the GWA discovery phase, several markers in the *OR2M4* (olfactory receptor, family 2, subfamily M, and member 4) gene region exhibited associations with p-values at the 10^−6^ level. The *OR2M4* is a small (~1 kb) single coding-exon gene located in the middle of a gene region that encompasses several olfactory receptor family 2 genes. We extended the genotyping markers to cover a broader list of nearby olfactory receptor family 2 genes in the fine-mapping study from *OR2M3* to *OR2T5*. Results of the set-based analysis also support the associations of the olfactory receptor family 2 genes and ASD. As recognizable by its name, the olfactory receptors interact with odorant molecules in the nose to initiate a neuronal response for smell. The olfactory system has long been studied to play important roles in establishing normal social behaviors and functions in mice [39]. In drosophila, olfactory communication is important for modulating aggressive behaviors in males through olfactory receptor neurons [40]. Patients with ASD, predominantly in males, are often characterized by impaired social interactions, inappropriate social communication, lack of emotional recognition, and increased aggressive behaviors. Notably, social communication difficulties are highly heritable. A recent GWA study of parent-reported social communication problems in a large UK population-based birth cohort found an association signal at 6p22.1 that encompasses an olfactory receptor gene cluster [41]. A prior genome-wide linkage scan for disinhibition of eating behaviors in a Quebec family study revealed a significant linkage peak on chromosome 19p13, where a cluster of seven olfactory receptor genes are located. Recently, Parma et al. (2013) reported that a mother's odor could promote social contact by increasing automatic imitation behaviors in children with autism, which provides extra evidence for the link between the olfactory system and the modulation of social behaviors in ASD. Additionally, the vasopressin system intrinsic to the olfactory system is found to play important roles in processing social information and regulating emotionality [42]. Although the olfactory receptor gene family does not appear as the top significant loci in a previous GWA study of ASD in Caucasian samples, the single marker and gene-based analysis results in our Taiwanese Han samples suggest the involvement of the olfactory system in the pathogenesis of ASD.

The other gene region that showed association with ASD in our fine-mapping study is in *GLIPR1/KRR1*. These two gene regions overlapped, and the most significant two markers in this region are rs3741496 (p-value = 1.15*10^−5^) and rs1051446 (p-value = 1.77*10^−5^). In gene-based testing, the *GLIPR1/KRR1* gene region exhibited a significant association with ASD in the GWA study (p-value = 3.5*10^−5^) as well as in the fine-mapping study (p-value = 0.015). Due to the partial overlap of the two genes, whether the effect of associated genetic variants on ASD is through which gene remains unclear. Increased expression of the *GLIPR1* (Glioma-GLI pathogenesis-related 1) gene is associated with myelomocytic differentiation in macrophage, and decreased expression of this gene through gene methylation is found to be associated with prostate cancer [43,44]. The *GLIPR1* gene is located in a cluster with two related genes on chromosome 12, including *GLIPR1L1* (gene-based p-value in the GWA stage was 1.8*10^−4^) and *GLIPR1L2* (gene-based p-value in the GWA stage was 1.8*10^−4^). Interestingly, a nonsense *de novo* mutation in the *GLIPR1L2* gene was found in schizophrenic patients [42]. On the other hand, the current understanding on the biological function of the human *KRR1* gene is relatively limited. It is a small subunit processome component that is required for the biogenesis of the 18S rRNA [45]. How exactly the *GLIPR1/KRR1* genes may be related to ASD remains questioned.

We found that several markers showed associations with ASD in the total samples after the Bonferroni’s correction (S2 Table). In particular, a few significant markers are located in the known gene regions of the *GLIS1* and *NAALADL2* genes. The *GLIS1* (GLI-similar 1) gene constitutes of a subfamily of the Kruppel-like zinc finger protein that functions as an activator and repressor of transcription [46]. This gene is reported to be associated with subclinical cardiovascular disease in African Americans with type 2 diabetes [47]. In addition, genetic variant (rs797906) in this gene is found to increase the risk for late-onset of Parkinson's disease in the Han Chinese population [48]. The patterns of gait disturbance and motor stereotypes in children with ASD have been interpreted using the Parkinsonian model [27,49,50]. These two stereotypes: hand/finger stereotypes (e.g., tapping, clapping, shaking, waving, opening-closing, and twirling the hands or fingers) and gait disturbances (e.g., skipping, spinning, pacing, jumping, and toe walking) have been discussed in the context of a “Parkinsonian” fronto-striatal basal-ganglia brain dysfunction [51]. Hence, further investigation of this genetic variant in another independent samples of ASD and Parkinsonian populations is warranted.

The *NAALADL2* (N-acetylated alpha-linked acidic dipeptidase-like 2) is a giant gene, which is implicated in a rare developmental malformation syndrome, the Cornelia de Lange syndrome. Patients with this syndrome are usually characterized by mental disability, growth retardation, distinctive facial features, and limb reduction defects [52]. Some of the clinical characteristics are also observed in a proportion of patients with ASD. In addition, one GWA study of Kawasaki disease, a pediatric vasculitis that damages coronary arteries, reported a marker (rs17531088, p = 1.13 x 10^−6^) in the *NAALADL2* gene associated with Kawasaki disease [53]. It seems that both the *GLIS1* and *NAALADL2* genes are involved in some forms of heart related health events—though the relationship between these genes with ASD needs further study.

The current study has several limitations. First, the current study might not have sufficient power to detect variants of small to moderate effects on traits. This might at least partly explain the initial GWA findings without any markers reaching genome-wide significance. Second, in the fine-mapping stage, we selected tagging markers for candidate genes based on HapMap results. It is still likely that we did not cover all the genetic variations in selected gene regions. Third, despite a few novel findings in the current study, our results of GWA case-control association study did not replicate common genetic findings of previous GWA studies in Caucasian samples. Ethnic heterogeneity might explain the differences, but again, the power issue is still a concern. Enlarging the sample size in the future is required to produce more robust genetic findings for ASD. Lastly, a genetic association study is prone to population stratification. Although we have tried to control such influences in the GWA stage using multiple-dimensional scaling analysis, and carefully recruited samples using the same inclusion criteria in both the discovery and additionally recruited samples, we may not be able to completely rule out potential influences by hidden subpopulations in our samples.

## Conclusions

In sum, we conducted the first GWA study of autism in the Taiwanese Han population with moderate sample size, and reported several genetic variants and gene regions associated with ASD. These new findings may lead to further investigation of the biological functions of specific genetic variants and gene regions for the risk of ASD. Despite these suggestive novel candidates for ASD, the underlying mechanisms and how these genetic variants may interact with other factors to influence the risks of developing ASD or even specific clinical syndromes are currently unknown. To replicate our findings in other independent samples locally or in other Asian countries is warranted before making any firm conclusions.

## Supporting Information

S1 Fig Study design flow diagram (PDF)

S2 Fig Quantile-quantile plot of pvalues (PNG)

S1 Table Haplotype analysis results with sliding window size 3 in the GWA study of 315 ASD cases and 1115 controls. (DOCX)

S2 Table Analysis for 20 markers that genotyped in the total samples. (DOCX)
