# Supplementary material for: Genome-Wide Association Study for Autism Spectrum Disorder in Taiwanese Han Population
Source: PLoS One. 2015 Sep 23;10(9):e0138695. doi: 10.1371/journal.pone.0138695 (PMC4580585; doi:10.1371/journal.pone.0138695)
Supplement: S1 Fig — (PDF) [file pone.0138695.s001.pdf]

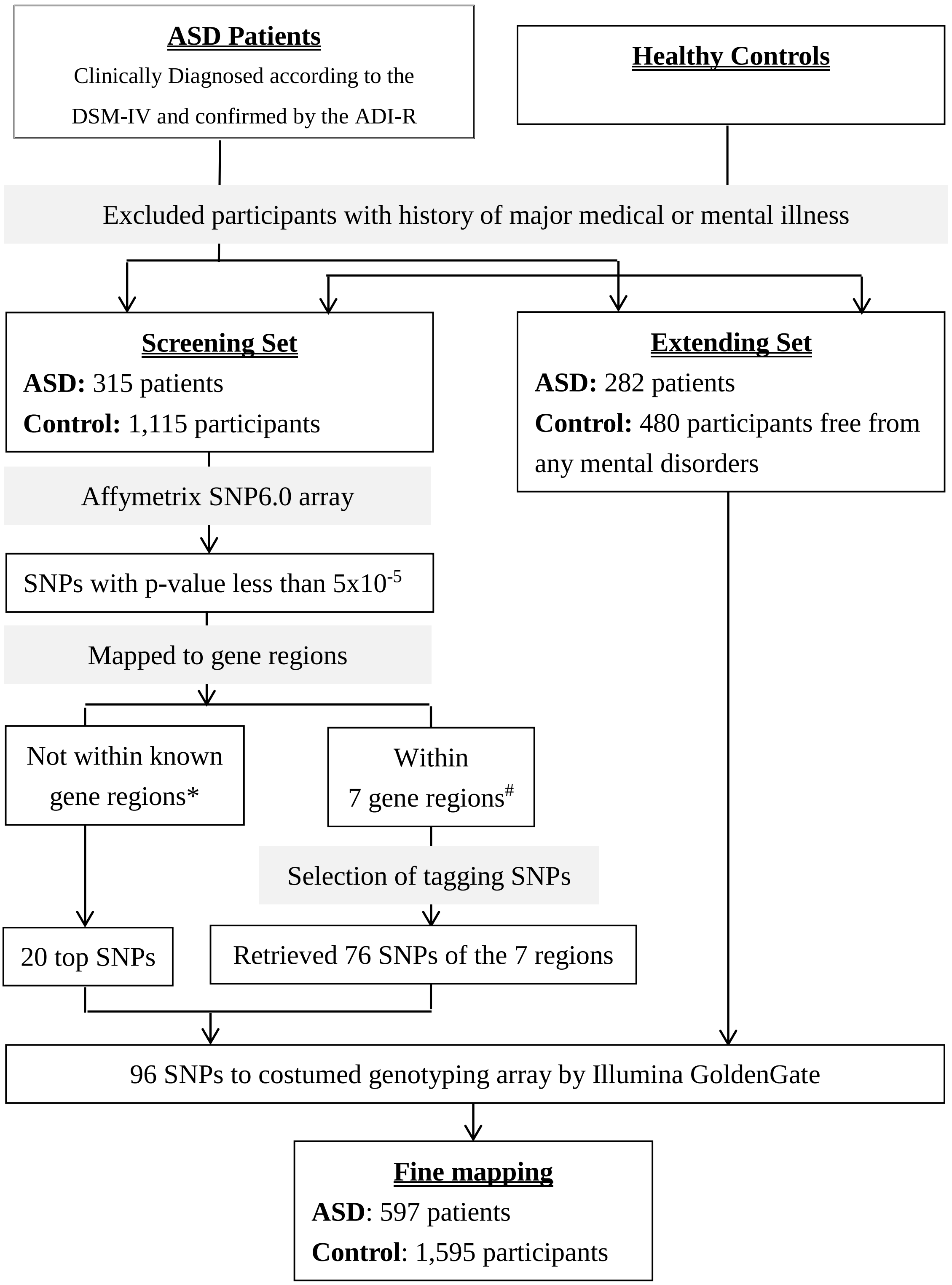

Supplementary Figure 1. Study design flow diagram

Abbreviation: DSM-IV: The Diagnostic and Statistical Manual of Mental Disorders, Fourth edition; **ADI-R:** The Autism Diagnostic Interview-Revised; **ASD:** autism spectrum disorder.

Including *OR2M3-OR2T5*, *STYK1*, *GSTZ1*, *GLIPR1-KRR1*, *DDX19A-DDX19B*, *SGSM2-MNT*, *KCNE1*.

***** One marker of a previously reported autism associated gene (*MACROD2*) with a p-value of $9.95*10^{-5}$ was also included.
