# Supplementary figures and images for: Genome-Wide Association Study for Autism Spectrum Disorder in Taiwanese Han Population
Source: PLoS One. 2015 Sep 23;10(9):e0138695. doi: 10.1371/journal.pone.0138695 (PMC4580585; doi:10.1371/journal.pone.0138695)

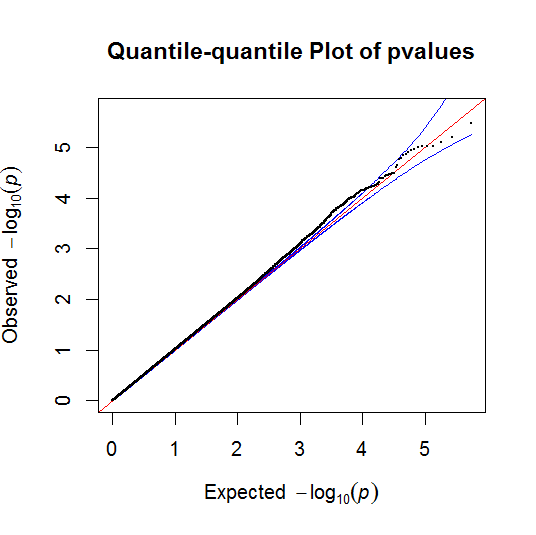

Supplement: S2 Fig — (PNG) [file pone.0138695.s002.png]
