# Supplementary material for: Genome-Wide Association Study for Autism Spectrum Disorder in Taiwanese Han Population
Source: PLoS One. 2015 Sep 23;10(9):e0138695. doi: 10.1371/journal.pone.0138695 (PMC4580585; doi:10.1371/journal.pone.0138695)
Supplement: S1 Table — (DOCX) [file pone.0138695.s003.docx]

**Supplementary Table 1. Haplotype analysis results with sliding window size 3 in the GWA study of 315 ASD cases and 1115 controls**

|  |  |  | **Nearby genes**  (MHF of ASD, MHF of controls) | |  | **MHF >0.05** | | |
| --- | --- | --- | --- | --- | --- | --- | --- | --- |
| **Chromosome** | **SNP** |  |  |  |  | **Chi_square** | **df** | **P-value** |
| **3** | **rs3849516-rs9873293-rs3914502** |  | ***NAALADL2*** | **(7kb away)** |  | **26.52** | **3** | **7.41X10^-06^** |
|  | GGT |  | (0.12, 0.20) | |  | 16.81 | 1 | 4.12X10^-05^ |
|  | GGA |  | (0.36,0.30) | |  | 9.13 | 1 | 2.52X10^-03^ |
|  | ACA |  | (0.48,0.45) | |  | 1.94 | 1 | 1.63X10^-01^ |
|  | GCA |  | (0.03,0.06) | |  | 5.66 | 1 | 1.73X10^-02^ |
| **17** | **rs2002863-rs2447097-rs2447095** |  | ***SGSM2/MNT*** | **(In gene)** |  | **17.29** | **2** | **1.76 X10^-04^** |
|  | CAA |  | (0.31,0.24) | |  | 13.78 | 1 | 2.06X10^-04^ |
|  | CCG |  | (0.08,0.12) | |  | 6.77 | 1 | 9.27X10^-03^ |
|  | TCG |  | (0.61,0.64) | |  | 2.73 | 1 | 9.87X10^-02^ |
| **20** | **rs6068777-rs290409-rs12479663** |  | ***CYP24A1/BCAS1*** | **(15-50kb away)** |  | **28.01** | **3** | **3.62X10^-06^** |
|  | CAG |  | (0.15,0.09) | |  | 19.57 | 1 | 9.68X10^-06^ |
|  | AAT |  | (0.16,0.13) | |  | 2.14 | 1 | 1.43X10^-01^ |
|  | CAT |  | (0.10,0.15) | |  | 8.70 | 1 | 3.18X10^-03^ |
|  | CGT |  | (0.59,0.63) | |  | 2.79 | 1 | 9.50X10^-02^ |

**MHF: Minor Haplotype Frequency**
