# Supplementary material for: Genome-Wide Association Study for Autism Spectrum Disorder in Taiwanese Han Population
Source: PLoS One. 2015 Sep 23;10(9):e0138695. doi: 10.1371/journal.pone.0138695 (PMC4580585; doi:10.1371/journal.pone.0138695)
Supplement: S2 Table — (DOCX) [file pone.0138695.s004.docx]

**Supplementary Table 2. Analysis for 20 markers that genotyped in the total samples**

|  |  |  |  | **Nearby** |  | **Total samples**  **(597 ASD/1,595 controls)** | | | | |
| --- | --- | --- | --- | --- | --- | --- | --- | --- | --- | --- |
| **CHR** | **SNP** | **Position** | **Allele** | **Genes** |  | **ASD** | **Control** | **OR** | **(95% C.I.)** | **P-value** |
| 1 | rs12082358 | 54,156,624 | C | ***GLIS1*** |  | 0.36 | 0.30 | 1.3 | (1.1-1.5) | 2.2X10^-04^ |
| 1 | rs12080993 | 54,178,501 | A | ***GLIS1*** |  | 0.35 | 0.29 | 1.3 | (1.1-1.5) | 1.5X10^-04^ |
| 2 | rs3916984 | 157,445,840 | A | *GPD2^a^* |  | 0.47 | 0.41 | 1.3 | (1.1-1.5) | 3.1X10^-04^ |
| 2 | rs13014164 | 170,271,231 | C | *LRP2/BBS5 ^a^* |  | 0.07 | 0.04 | 1.7 | (1.3-2.3) | 8.6X10^-05^ |
| 3 | rs3914502 | 174,564,602 | A | ***NAALADL2*** |  | 0.46 | 0.38 | 1.4 | (1.2-1.6) | 3.5X10^-06^ |
| 3 | rs2222447 | 174,580,826 | A | ***NAALADL2*** |  | 0.15 | 0.21 | 0.7 | (0.6-0.8) | 5.3X10^-05^ |
| 4 | rs7697680 | 55,245,541 | G | *PDGFRA ^a^* |  | 0.10 | 0.07 | 1.5 | (1.2-1.9) | 9.2X10^-04^ |
| 5 | rs11741756 | 132,579,659 | A | ***FSTL4*** |  | 0.15 | 0.12 | 1.3 | (1.1-1.5) | 1.2X10^-02^ |
| 6 | rs13211684 | 51,175,486 | G |  |  | 0.25 | 0.21 | 1.3 | (1.1-1.5) | 2.5X10^-03^ |
| 8 | rs12543592 | 123,511,652 | G |  |  | 0.42 | 0.50 | 0.7 | (0.6-0.8) | 3.2X10^-06^ |
| 9 | rs10966205 | 24,095,607 | T |  |  | 0.39 | 0.33 | 1.3 | (1.2-1.5) | 2.9X10^-05^ |
| 9 | rs7026342 | 111,280,201 | C |  |  | 0.09 | 0.06 | 1.6 | (1.2-2.0) | 1.8X10^-04^ |
| 9 | rs7030851 | 111,281,164 | A |  |  | 0.09 | 0.06 | 1.6 | (1.3-2.0) | 1.4X10^-04^ |
| 10 | rs10763893 | 33,011,229 | A | ***C10orf68*** |  | 0.07 | 0.04 | 1.6 | (1.2-2.2) | 6.1X10^-04^ |
| 11 | rs12366025 | 29,148,890 | A |  |  | 0.16 | 0.13 | 1.3 | (1.1-1.6) | 3.8X10^-03^ |
| 11 | rs11030597 | 29,190,528 | G |  |  | 0.16 | 0.13 | 1.3 | (1.1-1.6) | 4.1X10^-03^ |
| 11 | rs7933990 | 29,208,679 | A |  |  | 0.16 | 0.12 | 1.3 | (1.1-1.6) | 2.5X10^-03^ |
| 11 | rs11030606 | 29,214,412 | A |  |  | 0.16 | 0.13 | 1.3 | (1.1-1.6) | 5.6X10^-03^ |
| 20 | rs17263514 | 14,046,551 | A | ***MACROD2*** |  | 0.23 | 0.19 | 1.2 | (1.0-1.4) | 1.4X10^-02^ |
| 20 | rs12479663 | 52,708,752 | C | *BCAS1/CYP24A1^a^* |  | 0.13 | 0.09 | 1.5 | (1.3-1.9) | 4.0X10^-05^ |

**CHR:** chromosome; **SNP:** single nucleotide polymorphism; **OR (95% CI):** odds ratios and 95% confidence interval. Genes in bold: markers are in gene regions. ^a^ The nearby gene is within 100 kb away.
